# South West Obstetrical and Gynaecological Society

**Published:** 1992-08

**Authors:** 


### South Western Obstetrical and Gynaecological Society

### Meeting held at the Haven Hotel, Poole, 11 October 1991

### HORMONE REPLACEMENT THERAPY FOR FROZEN EMBRYO TRANSFER

Mr David W. Davies
Princess Anne Hospital, Southampton

The Southampton In Vitro Fertilisation Programme was established in 1986. Since that time the programme has maintained a clinical pregnancy rate of over 25 per oocyte retrieval. Embryos in excess of requirements for fresh transfer have been cryopreserved using controlled rate freezing and Propanediol as a cryoprotectant. Review of the first 75 frozen embryo transfers in natural or clomid induced cycles has produced only three pregnancies. A programme of pituitary down-regulation followed by preparation of the patient's endometrium with exogenous oestradiol valerate and progesterone was established in order to improve the success rate with cryopreserved embryos.

The patients were treated with Buserelin for three weeks prior to administration of 4-6mgs of oral oestradiol valerate daily. After 14 days treatment intramuscular progesterone 100mgs daily was introduced to the regime and embryo transfer was undertaken after the third progesterone injection. The initial pilot study produced five clinical pregnancies in 18 cycles. Analysis of patients' biochemical response suggested that the serum oestradiol achieved in the late proliferative phase was significantly higher in the group of patients who became pregnant. Further cycles were undertaken with the express aim of achieving a peak serum proliferative phase oestradiol level >800pmol/1. To date 55 embryo transfers have been undertaken, resulting in 14 clinical pregnancies (25.5%). Pregnancy has to be maintained for the first few weeks due to lack of corpus luteum function. Oestrogen therapy was maintained for an average of 67 days and progesterone therapy for 88 days. To date 11 of the pregnancies have delivered, including one set of twins, two are ongoing and there has been one spontaneous abortion at nine weeks gestation. There have been no recorded congenital anomalies.

Further work is continuing to optimise drug treatment regimes and to evaluate the use of vaginal rather than intramuscular progesterone administration.

### SCREENING FOR CIN — IS CYTOLOGY WORTHWHILE?

Mr John A Giles
Yeovil District Hospital, Yeovil, Somerset

Squamous cell carcinnoma of the cervix should be a preventable disease.If patients who have cervical intra-epithelial neoplasia are identified and treated, this should prevent the development of invasive disease.Cervical cytology has been used for many years as a screening process to identify these women. Over the last 10 years, increasing doubts have been raised as to the sensitivity and specificy of cervical cytology.

To investigate the accuracy of cervical cytology screening, a study was designed in which 200 normal women who had never had abnormal cytology were recruited. All patients had a cervical smear and were colposcoped. 11% of patients had a histological diagnosis of CIN. Cervical cytology identified all those patients with major grade disease and all those patients with large CIN lesions. Cervical cytology was not accurate in identifying small low-grade CIN lesions. The overall false-negative rate for cytology screening was 4%.

The natural history of small low-grade CIN lesions is unknown but it is unlikely that their identification (by colposcopy or cervicography) and treatment would be in the patients best interests. If these lesions prove to be of clinical significance, then as they increase in size or grade, the cytology will become abnormal and the patients at that point can be referred for treatment.

A second study was performed to look at the correlation between referral cytology and cervical disease. 200 women with mildly dyskaryotic smears were seen and colposcoped. One third of these patients as expected, had a CIN I lesion, one third of patients were normal and one third of patients had a CIN II or CIN III lesion. As the cervical cytology taken immediately prior to the colposcopic examination correlated well with the histological abnormality, it must be concluded that the lack of correlation between referral smear and histology is due to inadequate sampling at the time of the initial smear.

Human Papilloma Virus type 16 was of no clinical use in identifying patients with significant disease. 17% of women with normal cytology and a normal cervix were HPV 16 positive. 60% of women who had had an abnormal smear but had a normal cervix were also HPV 16 positive. The clinial significance of HPV 16 infection of the cervix is unknown.

A third colposcopic study on patients who had been treated for CIN showed that cytology had a false-negative rate of less than 4%.

In conclusion, it is safe to say that cervical cytology is an effective method of screening for CIN. The false-negative rate in a screened population is less than 4% and those lesions missed are small and of a low histological grade. The main source of error in cervical cytology screening is caused by inadequate sampling.

### THE TRIPLE OBSTETRIC TRAGEDY

Mr Kenneth Cooper
Worthing and Southlands Hospitals, Worthing

The antenatal care of Princess Charlotte in 1817 included a diet, episodes of venesection and sponging the loins with cold water. Labour began after 42 weeks and uterine action was poor throughout. After a first stage of 26 hours the second stage lasted 24 hours during which the obstetrician, Sir Richard Croft, was on the horns of a dilemma. The standards of the day were ultraconservative about the use of forceps subsequent to many deaths caused by the excessive use of force in previous years. A current text book, written by Croft's father-in-law, required the head to be six hours on the perineum and the absence of uterine contractions before forceps should be applied, and therefore Croft declined to use them. The Princess finally delivered spontaneously of a 91b stillborn male and required manual removal of the placenta; she became restless and dyspnoeic and died four hours after delivery. At autopsy a considerable volume of blood in the uterine cavity suggested death from circulatory failure and postpartum haemorrhage, perhaps aggravated by prepartum anaemia. Croft had followed the teaching of the day but took the misfortune much to heart and shot himself three months after the delivery, thus becoming the third member of the 'Triple Obstetric Tragedy'.

### SCREENING AND PREVENTION OF OVARIAN CARCINOMA

Dr Jane Bridges
Princess Anne Hospital, Southampton

Survival rates for ovarian cancer have not improved in the last three decades despite advances in surgical techniques and chemotherapeutic regimens. The investigation of possible screening techniques and preventative surgery for this disease is therefore of great importance.

The place of ultrasound has been extensively investigated by the Kings College Group. Over 3,000 post menopausal women were scanned at yearly intervals for three years. Eight cases of ovarian cancer were detected with a specificity of 96.4%. At the Royal London Hospital, Jacobs et al reported a 99.8% specificity using serum CA125 estimation with ultrasound as a secondary procedure. Following this initial prevalent screen of more than 21,000 post menopausal women a randomised incidence screening programme has been initiated. Although the specificity and sensitivity of the tumour marker CA125 may prove to be unacceptable as a screening technique the discovery of more sensitive markers, or the use of multiple markers, may be of use in the future.

Until a reliable screening technique is available the place of prophylactic oophorectomy should not be forgotten. The 0.2% prospective cancer risk figure quoted by many is invalid as many women in the studies responsible for this figure were not followed up until death. It is clear, however, that between 7-15% of women who develop ovarian cancer have undergone previous pelvic surgery when the ovaries could have been removed. With these figures in mind women undergoing abdominal hysterectomy should have appropriate counselling as to the place of prophylactic oophorectomy.

### UPDATE ON PRENATAL DIAGNOSIS

Mr Dominic L. Byrne
St Thomas' Hospital, London

The tremendous advances in all aspects of prenatal diagnosis over the last 15 years have not reduced the proportion of chromosomally abnormal births in this country. The reason for this paradox is that maternal age is a crude risk marker, as only 5% of the obstetric population are considered high risk, and they produce only 30% of the affected pregnancies.

Biochemical triple screening offers the possibility of detecting 60% of Trisomy 21 pregnancies, providing screening is applied to the whole obstetric population. It must be appreciated therefore, that all mothers are at risk and that this test will adjust the risk for an individual pregnancy, guiding whether an invasive procedure is required or not. By applying invasive procedures in cases of high risk and not advanced maternal age the detection rate will be improved, and the number of procedures not be increased.

Ultrasound can also be used for screening, by detecting foetal chromosomal markers at 20 weeks, and more recently by using nuchal translucency as a marker at 10-12 weeks.

The main invasive procedures of amniocentesis and chorion villus sampling have been joined by the new techniques of early amniocentesis and amnifiltration, which are present being evaluated for their safety and diagnostic accuracy.

### DOCTOR AT SEA — A GP TRAINEE'S VIEW OF THE GULF CONFLICT

Surgeon Lt-Cdr. Andrew Newton
Royal Naval College, Greenwich

HMS CARDIFF - The present CARDIFF is the third ship to bear the name.The first was an 18 gun French Privateer captured in the North Sea in 1652 which remained in service for only six years before being sold.

The second was a light cruiser of 4190 tons built by Fairfields on the Clyde, and launched in 1917. She was armed with 5 x 6 inch guns and had a top speed of 29 knots. She took part in the battle of Heligoland Bight in 1917 as flagship of the 6th light cruiser squadron, and earned her place in history by leading the German High Seas Fleet to surrender in the Firth of Forth on 21 November 1918. She saw active service worldwide up to the start of World War 2 when she became a gunnery training ship in the Clyde before being broken up in 1946.

The current HMS CARDIFF was built by Vickers at Barrow and launched by Lady Caroline Gilmore on 22 February 1974. Her fitting out was delayed by industrial action until she was eventually towed to Swan Hunter on the Tyne to be completed. The ship was finally commissioned on 24 September 1979.

**Facts and Figures**

| | |
|---|---|
| Length: | 125 metres |
| Beam: | 14.6 metres |
| Displacement: | 3,560 tons |
| Complement: | 290 |
| Armament: | Sea Dart Missile System / Mark 8 114mm Gun / Phalanx CIWS |
| Propulsion: | Rolls Royce Olympus Gas Turbines (x 2) : Tyne Gas Turbines (x 2) |
| Maximum Speed: | 28 knots |

**THE STATISTICS OF WAR...**

**Seamanship**

| | |
|---|---|
| * Total distance steamed | 29,367 Nautical Miles |
| * Total number of days away from Portsmouth | 165 |

**SUPPLY**

| | |
|---|---|
| * Total quantities of food consumed | 49,550 Eggs<br>36, 355 LBS of Vegetables<br>3,211 LBS of Mince |

...All of this and each and every one of us still slept for over 25% of the deployment !

